# Construction of a cancer-associated fibroblasts-related long non-coding RNA signature to predict prognosis and immune landscape in pancreatic adenocarcinoma

**DOI:** 10.3389/fgene.2022.989719

**Published:** 2022-09-23

**Authors:** Yingquan Ye, Qinying Zhao, Yue Wu, Gaoxiang Wang, Yi Huang, Weijie Sun, Mei Zhang

**Affiliations:** ^1^ Oncology Department of Integrated Traditional Chinese and Western Medicine, The First Affiliated Hospital of Anhui Medical University, Hefei, China; ^2^ The Traditional and Western Medicine (TCM)-Integrated Cancer Center of Anhui Medical University, Hefei, China; ^3^ Department of Infectious Diseases, The First Affiliated Hospital of Anhui Medical University, Hefei, China

**Keywords:** cancer-associated fibroblasts, pancreatic adenocarcinoma, lncRNA, prognostic, immune

## Abstract

**Background:** Cancer-associated fibroblasts (CAFs) are an essential cell population in the pancreatic cancer tumor microenvironment and are extensively involved in drug resistance and immune evasion mechanisms. Long non-coding RNAs (lncRNAs) are involved in pancreatic cancer evolution and regulate the biological behavior mediated by CAFs. However, there is a lack of understanding of the prognostic signatures of CAFs-associated lncRNAs in pancreatic cancer patients.

**Methods:** Transcriptomic and clinical data for pancreatic adenocarcinoma (PAAD) and the corresponding mutation data were obtained from The Cancer Genome Atlas database. lncRNAs associated with CAFs were obtained using co-expression analysis. lncRNAs were screened by Cox regression analysis using least absolute shrinkage and selection operator (LASSO) algorithm for constructing predictive signature. According to the prognostic model, PAAD patients were divided into high-risk and low-risk groups. Kaplan-Meier analysis was used for survival validation of the model in the training and validation groups. Clinicopathological parameter correlation analysis, univariate and multivariate Cox regression, time-dependent receiver operating characteristic (ROC) curves, and nomogram were performed to evaluate the model. The gene set variation analysis (GSVA) and gene ontology (GO) analyses were used to explore differences in the biological behavior of the risk groups. Furthermore, single-sample gene set enrichment analysis (ssGSEA), tumor mutation burden (TMB), ESTIMATE algorithm, and a series of immune correlation analyses were performed to investigate the relationship between predictive signature and the tumor immune microenvironment and screen for potential responders to immune checkpoint inhibitors. Finally, drug sensitivity analyses were used to explore potentially effective drugs in high- and low-risk groups.

**Results:** The signature was constructed with seven CAFs-related lncRNAs (AP005233.2, AC090114.2, DCST1-AS1, AC092171.5, AC002401.4, AC025048.4, and CASC8) that independently predicted the prognosis of PAAD patients. Additionally, the high-risk group of the model had higher TMB levels than the low-risk group. Immune correlation analysis showed that most immune cells, including CD8^+^ T cells, were negatively correlated with the model risk scores. ssGSEA and ESTIMATE analyses further indicated that the low-risk group had a higher status of immune cell infiltration. Meanwhile, the mRNA of most immune checkpoint genes, including PD1 and CTLA4, were highly expressed in the low-risk group, suggesting that this population may be “hot immune tumors” and have a higher sensitivity to immune checkpoint inhibitors (ICIs). Finally, the predicted half-maximal inhibitory concentrations of some chemical and targeted drugs differ between high- and low-risk groups, providing a basis for treatment selection.

**Conclusion:** Our findings provide promising insights into lncRNAs associated with CAFs in PAAD and provide a personalized tool for predicting patient prognosis and immune microenvironmental landscape.

## Introduction

Pancreatic cancer is highly malignant with a poor prognosis, leading to almost as many deaths as cases of its incidence, and is the seventh leading cause of cancer death (Sung et al., 2021). Pancreatic adenocarcinoma (PAAD) is the most common type of pancreatic cancer. A study from 28 EU countries predicts that pancreatic cancer will be the third leading cause of cancer deaths by 2025 (Ferlay et al., 2016). Given this grim situation, it is imperative to identify the prognostic signatures of PAAD patients and stratify and precisely treat them to improve the accuracy of prognostic judgments and the efficacy of individualized treatment, and improve prognosis.

A large amount of stroma constituting dense mesenchyme is the main feature of the PAAD tumor microenvironment (TME). Cancer-associated fibroblasts (CAFs) are one of the essential stromal components involved in multiple stages of tumor development through different pathways (Hinshaw and Shevde, 2019; Joshi et al., 2021). CAFs-derived chemokines, cytokines, exosomes, and growth factors not only promote tumor proliferation but also alter the immune cell environment by recruiting immunosuppressive cells and inhibiting the activity of immune effector cells to induce immune evasion of cancer cells (Tang et al., 2012; Martinez-Outschoorn et al., 2014; Öhlund et al., 2017; Kobayashi et al., 2019). In addition, CAFs promote the expression of immune checkpoint molecules and extracellular matrix remodeling (Harper and Sainson, 2014; Sun et al., 2018), thus indirectly affecting the activity of immune cells in the tumor immune microenvironment (TIME). Therefore, the interaction between CAFs and immune cells is vital in regulating TIME in pancreatic cancer.

Long non-coding RNAs (lncRNAs) are non-coding RNAs with more than 200 nucleotides. They are used as cancer biomarkers for diagnosis and prognosis since they can be dynamically monitored at different disease phases and better represent various cancer features (Yan et al., 2015). Studies show that lncRNAs can regulate gene expression in different transcriptional states and epigenetic processes, mediating tumor angiogenesis and immune escape (Zhao et al., 2018; Dragomir et al., 2020). lncRNAs are also widely involved in the growth, invasion, migration, and prognosis of pancreatic cancer (Gong and Jiang, 2020; Ramya Devi et al., 2021; Takahashi et al., 2021). However, the application of CAFs-related lncRNAs in predicting prognosis and immune microenvironment of PAAD patients is yet to be understood.

This study constructed a CAFs-associated lncRNA signature to stratify PAAD patients by risk status to predict prognosis and TIME characteristics. It also provides a reference for selecting individualized treatment options such as immune checkpoint inhibitors (ICIs) and targeted drugs.

## Materials and methods

### Data collection

Transcriptome expression profiles, mutation data, and relevant clinical information of patients with PAAD were obtained from The Cancer Genome Atlas (TCGA) database (https://portal.gdc.cancer.gov/repository). The data were then collated using Strawberry Perl (version 5.32.1.1) scripts to obtain mRNA and lncRNA data matrixes for subsequent studies. The 86 CAFs related genes used for the study were obtained from The Human Gene Database (https://www.genecards.org/), with a relevance score of >11 (Supplementary Appendix S1).

### Identification of cancer-associated fibroblasts-related long non-coding RNAs

The mRNA expression data of 86 CAFs-related genes were extracted using the R package “limma.” The set of CAFs-related lncRNAs was obtained by co-expression analysis using a Pearson correlation coefficient >0.4 and a threshold of *p* < 0.001. The correlation data of CAFs-related genes and lncRNAs were constructed using the R packages “dplyr,” “ggalluvial,” and “ggplot2,” and correlation Sankey plots were generated. Subsequently, the “limma” package was used to merge the survival and lncRNA expression data of each PAAD patient.

### Establishing a risk model based on the cancer-associated fibroblasts-related long non-coding RNAs signature

The R packages “caret,” “timeROC,” “survminer,” “survival,” and “glmnet”were used to establish the risk signature of CAFs-related LncRNAs in PAAD. Data of the patients obtained from the TCGA were randomly divided into training and validation groups by 1:1, and various clinical traits were analyzed in the two groups. Then the prognosis-associated lncRNAs in the training group were obtained using univariate Cox analysis, and the prognostic forest plots (*p* < 0.05) were plotted. Subsequently, the “pheatmap” package was used to plot the expression heat map of prognosis-related lncRNAs. The LASSO regression analysis was used to screen candidate lncRNAs to avoid overfitting. Risk scores for all patients were obtained by the following formula: risk score = (coefficient lncRNA1*lncRNA1 expression) + (coefficient lncRNA2*lncRNA2 expression) + …+ (coefficient lncRNAn* lncRNAn expression). Coefficient and expression represent the regression coefficient and expression values of the CAFs-related lncRNAs model, respectively. Based on the median risk score in the training cohort, patients in the validation and training cohorts were split into high- and low-risk groups. In addition, the R packages “tidyverse,” “ggplot2,” and “ggExtra” were used for expression correlation analysis of CAFs-related genes and model lncRNAs and to draw a correlation heat map. The “limma,” “survivor,” and “survminer” packages were used to plot Kaplan-Meier (KM) curves for the high- and low-expression groups of model lncRNAs.

### Validation of the risk model

The R packages “pheatmap,” “survival,” and “survminer” were used for survival analysis of the training, validation, and entire cohorts. Risk curves, risk heat maps, and survival status maps were plotted for each cohort. In addition, overall survival (OS) and progression-free survival (PFS) survival curves were also plotted for the all cohort. Univariate and multivariate Cox regression analyses were used to assess whether the risk score and selected clinical characteristics were independent prognostic factors. Additionally, ROC curve analyses were performed using “survminer”, “survival”, and “timeROC” to assess the prognostic value of the developed signature by the area under the curve. Furthermore, the “survival,” “rms,” and “pec” packages were used to calculate the consistency index (c-index) to evaluate the best prediction of the model.

### Correlation analysis of the signature with clinicopathological parameters

The R package “ComplexHeatmap” was used to plot the heat map of the relationship between the high- and low-risk groups of the model and different clinicopathological parameters. Meanwhile, “survminer” and “survival” were used to plot the survival curves of high- and low-risk groups to determine whether the constructed risk model applied to PAAD patients with different clinicopathological parameters.

### Nomogram construction

Based on the results of the multivariate regression analysis, risk status and age were used to construct nomograms for 1-year, 3-years, and 5-years OS using the “regplot,” “survival,” and “rms” R packages. Hosmer-Lemeshow test calibration curve (method = “boot”, B = 1,000) was utilized to validate if the actual results correlate with the anticipated results.

### Functional analysis and mechanism exploration

The Gene Set Variation Analysis (GSVA) computationally detects differences in pathway activity in a sample population (Hänzelmann et al., 2013). GSEA enrichment analysis by the R package “limma,” “GSEABase,” and “GSVA” to obtain the enrichment of the Kyoto Encyclopedia of Genes and Genomes (KEGG) pathway in the high-risk and low-risk groups. The R package “pheatmap” was used to plot the pathway enrichment heatmap. In addition, differentially expressed genes between high- and low-risk populations (A fold change >2 and FDR <0.05) were identified using the R package “limma.” Subsequently, “enrichplot,” “GOplot,” “ggplot2,” “org.Hs.eg.db,” and “clusterProfiler” packages were used to complete GO analysis and explore potential pathways.

### Correlation between cancer-associated fibroblasts-related long non-coding RNAs signature and tumor mutation burden

The Strawberry Perl script collated the PAAD mutation data downloaded from the TCGA to obtain the tumor mutation burden (TMB) data for each patient. R packages “Limma” and “ggpubr” were used to analyze the TMB differences between the high- and low-risk groups of the model and generate violin plots. Meanwhile, “maftools” was used to map the mutation waterfall of the 15 genes with the highest mutation frequency in the high- and low-risk groups. In addition, the R software was used to obtain the optimal cutoff values of tumor mutation burden and classify patients into low-TMB and high-TMB groups. The “survivor” and “survminer” packages were used to plot the survival curves of patients in two risk groups and the survival curves of patients in the high- and low-TMB groups combined with the high- and low-risk groups.

### Correlation between cancer-associated fibroblasts-related long non-coding RNAs signature and immune microenvironment

CAFs play an essential regulatory role in the tumor immune microenvironment. To further explore the correlation between risk models constructed by CAFs-related lncRNA signature and immune microenvironment, the R packages “ggtext,” “tidyverse,” “ggpubr,” “scales,” and “ggplot2” were used to analyze the correlation between immune cells and risk scores and generate correlation bubble plots. In addition, single sample GSEA (ssGSEA) was performed to classify gene sets with common physiological regulation and biological functions (Subramanian et al., 2005). The “GSEABase” and “GSVA” packages were utilized for ssGSEA analysis to calculate the immune cell and immune-related function scores of the samples. “reshape2,” “ggpubr”, “pheatmap,” and “reshape2” packages were used to obtain box plots for ssGSEA differential analysis and heat maps for immune function differences in high- and low-risk groups of the model.

The potential predictive value of CAFs-related lncRNA signature for immune checkpoint efficacy was explored by utilizing the “ggplot2” and “ggpubr” packages to analyze the differences of immune checkpoint-related genes between the high- and low-risk groups and generate box plots of differentially expressed genes. Immune checkpoint programmed death-ligand 1 (PD-L1) on cancer cells binds to programmed cell death-1 (PD-1) on immune cells and contributes to the immune escape of tumor cells (Yi et al., 2021). Furthermore, in some cases, tumor PD-L1 expression correlates with immunotherapy response (Yu et al., 2016; Yi et al., 2018). “ggpubr” and “limma” packages analyzed the correlation between PD-L1, PD1, and CTLA4 expression and seven model lncRNAs and “corrplot” package plotted the correlation.

ESTIMATE is a new algorithm for counting immune and stromal cells infiltrating tumor tissue (Yoshihara et al., 2013). Using the R packages “ESTIMATE” and “limma,” the amount of immune and stromal cells in the tumor tissue of each PAAD case was evaluated to determine the corresponding scores. The sum of the stromal and immune scores is the ESTIMATE score, which is inversely linked to tumor purity. Box plots illustrating the differences in stromal, immune, and ESTIMATE scores between high- and low-risk groups were generated using the “ggpubr” package.

### Drug sensitivity analysis

The potential clinical significance of CAFs-related lncRNA signatures in chemotherapy and targeted therapies were explored using the half-maximal inhibitory concentrations (IC50) of different drugs in the high-risk and low-risk groups, obtained using the R packages “ggpubr” and “pRRophetic” (Geeleher et al., 2014). Drugs with different IC50s in the two groups were represented as box plots (*p* < 0.001).

## Results

### Cancer-associated fibroblasts-related long non-coding RNAs in pancreatic adenocarcinoma

**FIGURE 1:**
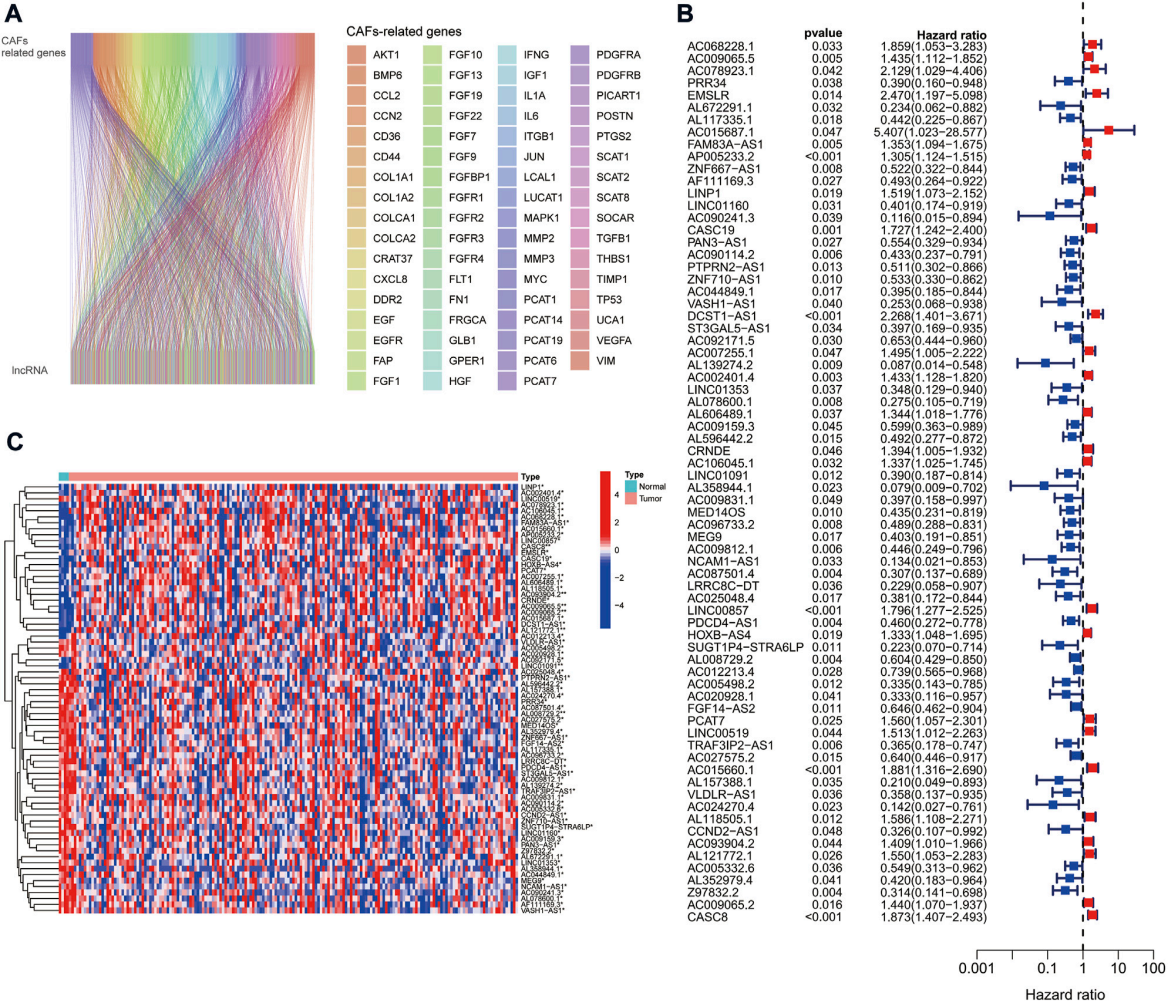
CAFs-associated lncRNAs in PAAD. **(A)** Sankey co-expression network plot of CAFs-associated genes and lncRNAs. **(B)** Prognostic forest plots showing the 72 CAFs-associated lncRNAs extracted by univariate Cox regression analysis. **(C)** Heat map of the 72 CAF-associated lncRNAs expression in normal and tumor samples.

We obtained expression data from TCGA database for 179 PAAD tumor samples. By co-expression analysis of mRNAs of CAFs-related genes, we obtained 378 CAFs-associated lncRNAs (correlation coefficient >0.4, *p* < 0.001) (Figure 1A).

### Construction and validation of cancer-associated fibroblasts-related long non-coding RNAs signature

**FIGURE 2:**
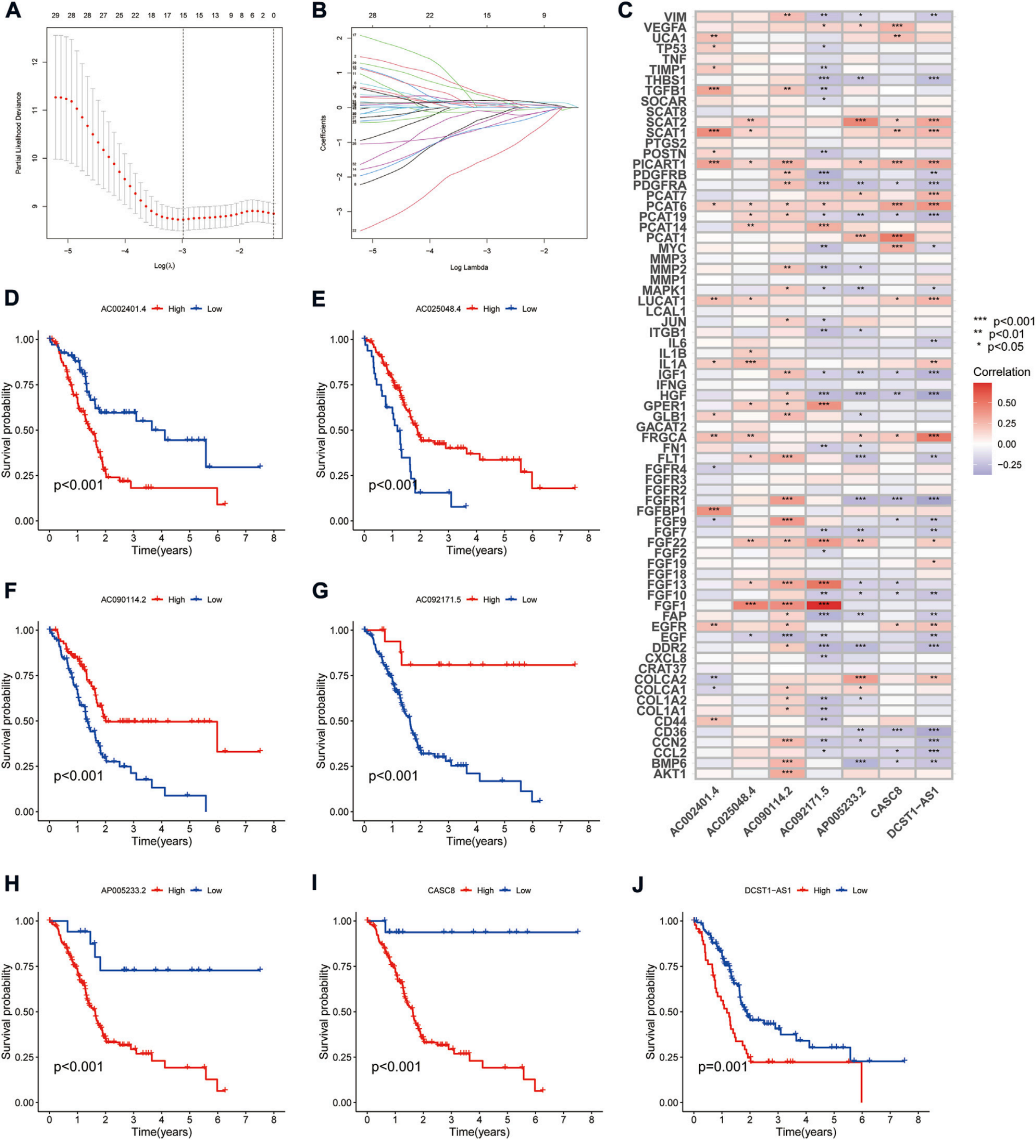
Derivation and selection of the CAFs-associated lncRNAs signature in the training cohort. **(A,B)** LASSO coefficient and partial likelihood deviance of the prognostic signature. **(C)** Heat map showing the correlation between the expression of seven lncRNAs and CAFs-related genes. **(D–J)** Kaplan-Meier curves analyzed the correlation between the expression of the seven crucial lncRNAs and the prognosis of PAAD.

The TCGA cohort was divided into training and validation groups, and clinicopathological characteristics were compared (Table 1). Univariate Cox (uni-Cox) regression analysis obtained 72 CAFs-related lncRNAs in the training group that was significantly associated with the OS of patients (*p* < 0.05). Figures 1B,C show the prognostic forest plot and expression heat map of the 72 LncRNAs, respectively. We further performed lasso regression analysis (Figures 2A,B) and extracted seven of these LncRNAs for model construction (Table 2). Risk scores were calculated from lncRNAs screened by lasso regression to create the formula: Risk score = AP005233.2 × (0.257) + AC090114.2 × (−0.829) + DCST1-AS1 × (0.596) + AC092171.5 × (−0.561) + AC002401.4 × (0.215) + AC025048.4 × (−1.388) + CASC8 × (0.348). The correlation between the expression of seven lncRNAs and CAFs-related genes was demonstrated with a heat map (Figure 2C).

**TABLE 1:** Comparison of clinicopathological features between the validation and training cohorts.

Covariates	Type	Total	Validation cohort	Training cohort	*p*-value
Age	≤65	94 (52.81%)	48 (53.93%)	46 (51.69%)	0.8807
	>65	84 (47.19%)	41 (46.07%)	43 (48.31%)	
Gender	FEMALE	80 (44.94%)	47 (52.81%)	33 (37.08%)	0.0501
	MALE	98 (55.06%)	42 (47.19%)	56 (62.92%)	
Grade	G1-2	126 (70.79%)	61 (68.54%)	65 (73.03%)	0.7997
	G3-4	50 (28.09%)	27 (30.34%)	23 (25.84%)	
	unknown	2 (1.12%)	1 (1.12%)	1 (1.12%)	
Stage	Stage I-II	168 (94.38%)	86 (96.63%)	82 (92.13%)	0.9631
	Stage III-IV	7 (3.93%)	3 (3.37%)	4 (4.49%)	
	Unknown	3 (1.69%)	0 (0%)	3 (3.37%)	
T	T1-2	31 (17.42%)	14 (15.73%)	17 (19.1%)	0.6416
	T3-4	145 (81.46%)	75 (84.27%)	70 (78.65%)	
	Unknown	2 (1.12%)	0 (0%)	2 (2.25%)	
M	M0	80 (44.94%)	45 (50.56%)	35 (39.33%)	1
	M1	4 (2.25%)	2 (2.25%)	2 (2.25%)	
	Unknown	94 (52.81%)	42 (47.19%)	52 (58.43%)	
N	N0	49 (27.53%)	24 (26.97%)	25 (28.09%)	0.886
	N1	124 (69.66%)	64 (71.91%)	60 (67.42%)	
	Unknown	5 (2.81%)	1 (1.12%)	4 (4.49%)	

**TABLE 2:** Long non-coding RNA signature models associated with CAFs.

CAFsLncSig	Coef	HR	HR (95%CI)	*p*-value
AP005233.2	0.257	1.305	1.124–1.515	<0.001
AC090114.2	−0.829	0.433	0.237–0.791	0.006
DCST1-AS1	0.596	2.268	1.401–3.671	<0.001
AC092171.5	−0.561	0.653	0.444–0.960	0.030
AC002401.4	0.215	1.433	1.128–1.820	0.003
AC025048.4	−1.388	0.381	0.172–0.844	0.017
CASC8	0.348	1.873	1.407–2.493	<0.001

HR, hazard ratio; CI, confidence interval.

**FIGURE 3:**
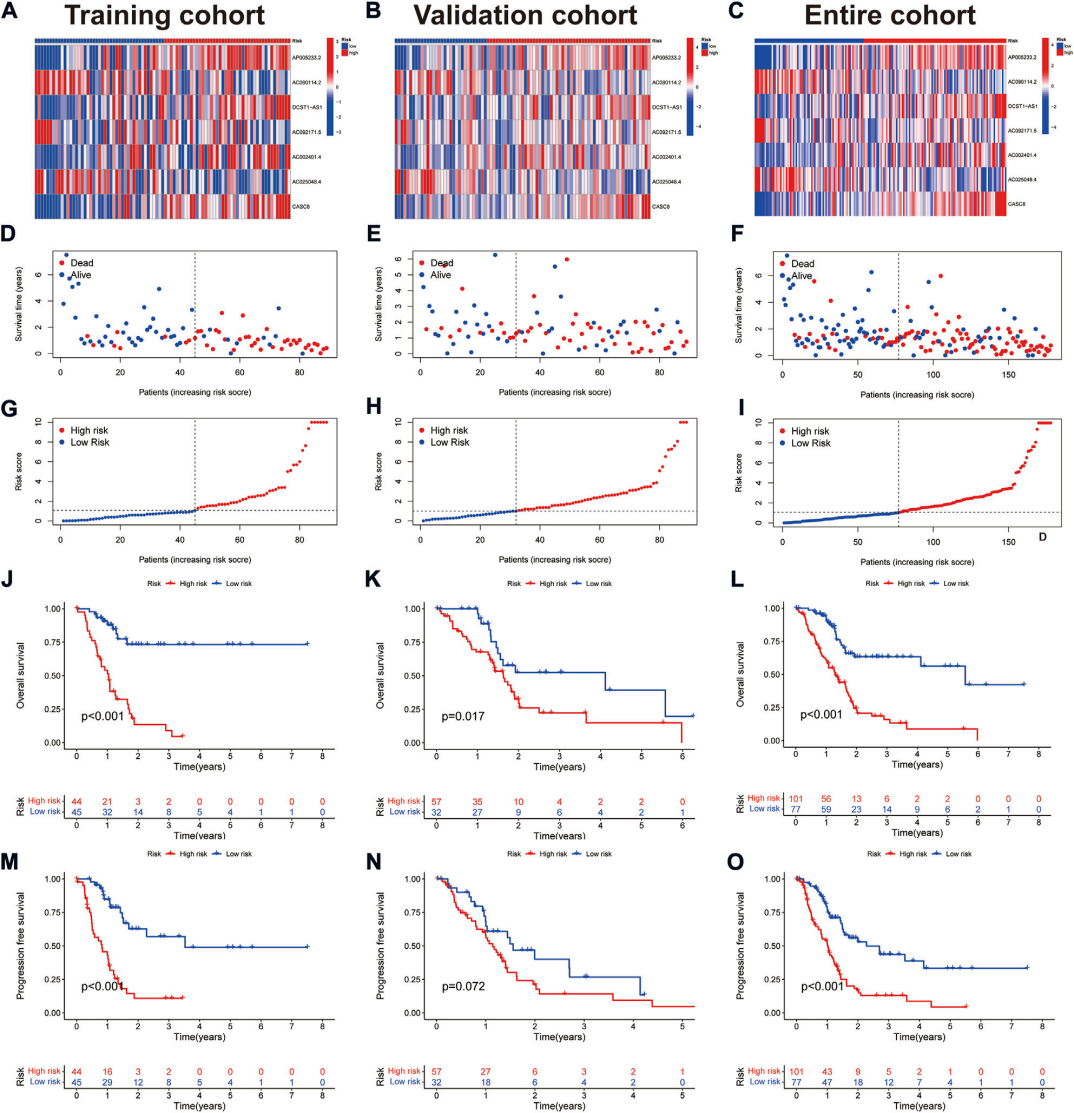
Prognostic values of the CAFs-associated lncRNAs signature. **(A–C)** Heat map showing expression levels of the seven lncRNAs in the training, validation, and entire cohorts. **(D–F)** Survival time and status in the training, validation, and entire cohorts. **(G–I)** Risk score distribution in the training, validation, and entire cohorts. **(J–L)** Kaplan-Meier curve for OS in the training, validation, and entire cohorts. **(M–O)** Kaplan-Meier curve for PFS in the training, validation, and entire cohorts.

Survival analysis showed that patients with high expression of AC090114.2, AC092171.5, and AC025048.4 had significantly better survival rates than the low expression group, while the remaining four genes showed the opposite effect (Figures 2D–J). The expression heat map showed that AC090114.2, AC092171.5, and AC025048.4 were low in both the training, validation, and entire cohorts in the high-risk group. Whereas consistent with the above results, the remaining four genes showed the opposite effect (Figures 3A–C). Subsequently, the survival status, risk score distribution, and OS survival curves of the low- and high-risk patients in the training, validation, and entire cohorts were assessed using the risk scores. All results showed that the prognosis of the low-risk group was significantly better than that of the high-risk group (Figures 3D–L). Similar results were obtained for the PFS survival curves of the training and entire TCGA cohorts. Although there was no statistical difference in PFS between the high- and low-risk groups in the validation group, a trend towards a separation of survival curves was observed (Figures 3M–O).

### Assessment of the cancer-associated fibroblasts-related long non-coding RNA signature

**FIGURE 4:**
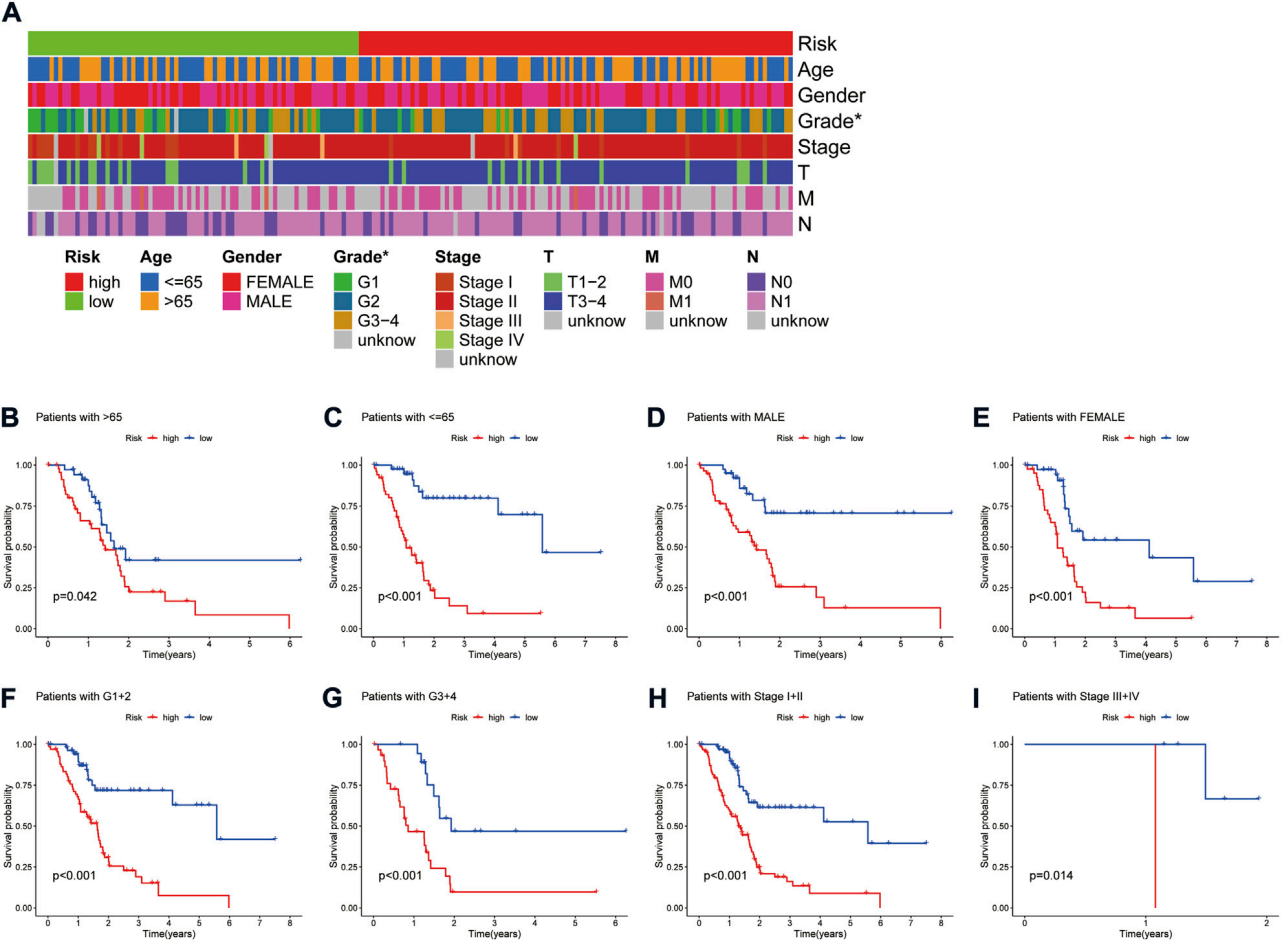
Correlation analysis of the risk signature with clinicopathological parameters. **(A)** Heat map of the distribution of clinicopathological parameters in the high- and low-risk groups. **(B,C)** Kaplan-Meier survival curves of low- and high-risk groups sorted by age. **(D,E)** Kaplan-Meier survival curves of low- and high-risk groups sorted by gender. **(F,G)** Kaplan-Meier survival curves of low- and high-risk groups sorted by grade. **(H,I)** Kaplan-Meier survival curves of low- and high-risk groups sorted by TNM stage.

**FIGURE 5:**
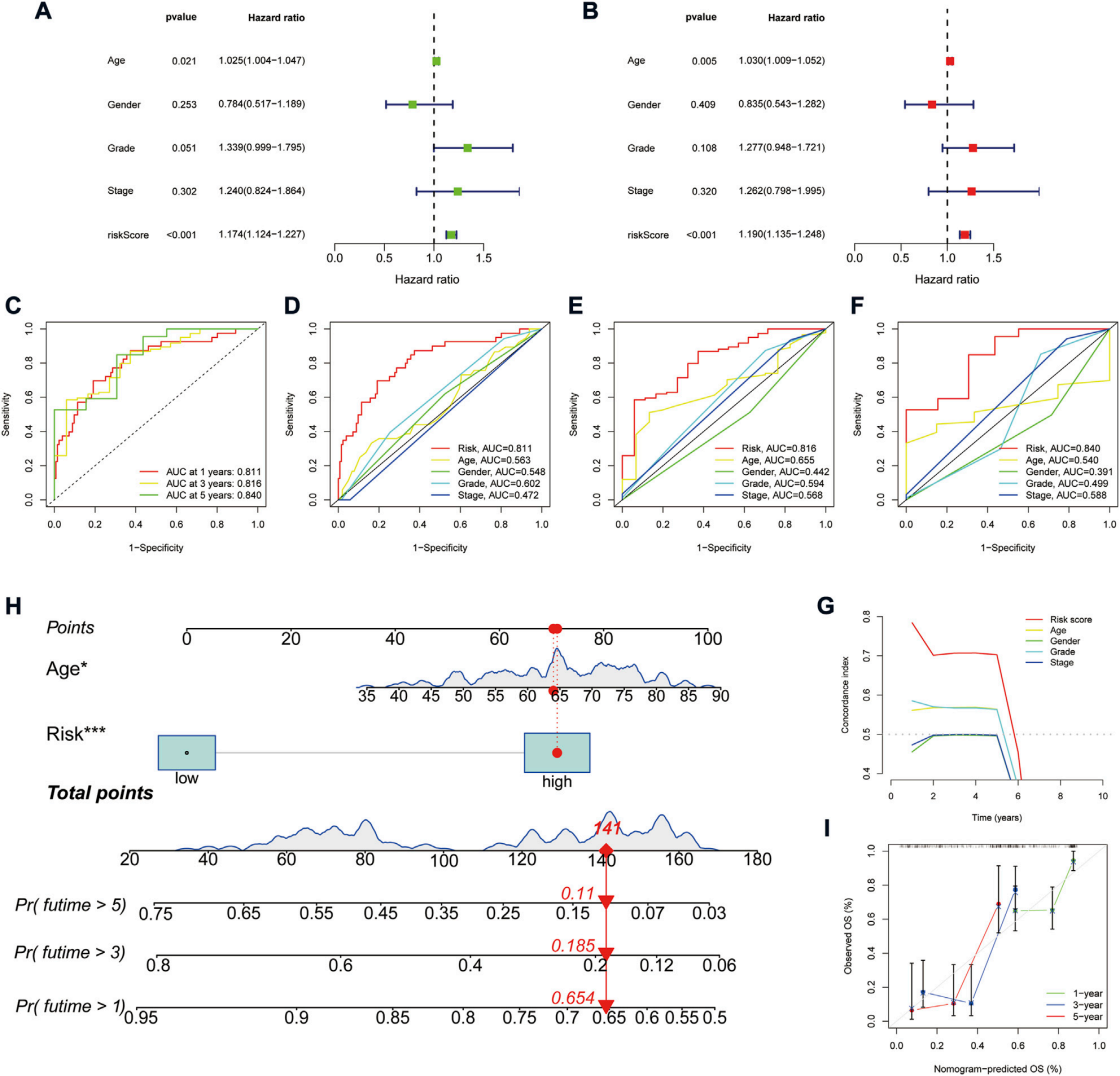
Assessment of the predictive signature. **(A,B)** Forest plot for univariate Cox **(A)** and multivariate Cox regression analysis **(B)**. **(C)** ROC curves of 1, 3, and 5 years survival for the predictive signature. **(D–F)** Comparison of the prediction accuracy of the risk model with age, gender, grade, and TNM stage at 1, 3, and 5 years. **(G)** The consistency index analysis curve. **(H)** Nomogram for predicting the 1, 3, and 5 years survival of patients with HCC. **(I)** The calibration curves for 1, 3, and 5 years OS.

A heat map of clinicopathological parameters showed differences in tumor grade between high- and low-risk groups (Figure 4A). Survival analysis showed that PAAD patients with different gender, ages, tumor grades, and stages all survived significantly better in the low-risk group than in the high-risk group (Figures 4B–I), demonstrating the model’s applicability to PAAD patients with different clinicopathological parameters. Further, univariate Cox (uni-Cox) regression and multivariate Cox (multi-Cox) regression suggested the risk score as an independent prognostic factor with hazard ratios (HR) of 1.174 and 1.190, respectively, with 95% confidence intervals (CI) of 1.124–1.227 (*p* < 0.001) and 1.135–1.248 (*p* < 0.001) (Figures 5A,B). Also, the patient’s age was an independent prognostic parameter. In addition, the ROC curve was used to assess the sensitivity and specificity of the risk model to the prognosis of PAAD. The results showed that the model had a significantly higher predictive value than other clinicopathological parameters, with an area under the curve (AUC) of 0.811, 0.816, and 0.840 at 1, 3, and 5 years respectively (Figures 5C–F). Furthermore, the c-index of the risk score was also higher than that of the other clinical parameters (Figure 5G). Together, these results demonstrate the good performance of the risk model.

### Nomogram construction

We constructed a nomogram based on the patient’s age and risk status to facilitate prognosis prediction for PAAD patients (Figure 5H). The corresponding scores for the patient’s age and risk status were calculated in the nomogram, and the total score was used as a prognostic prediction tool. A calibration curve was also plotted (Figure 5I). The results showed a good agreement between the survival of the PAAD patients and the values predicted by the nomogram.

### Gene set variation analysis and gene ontology analysis

**FIGURE 6:**
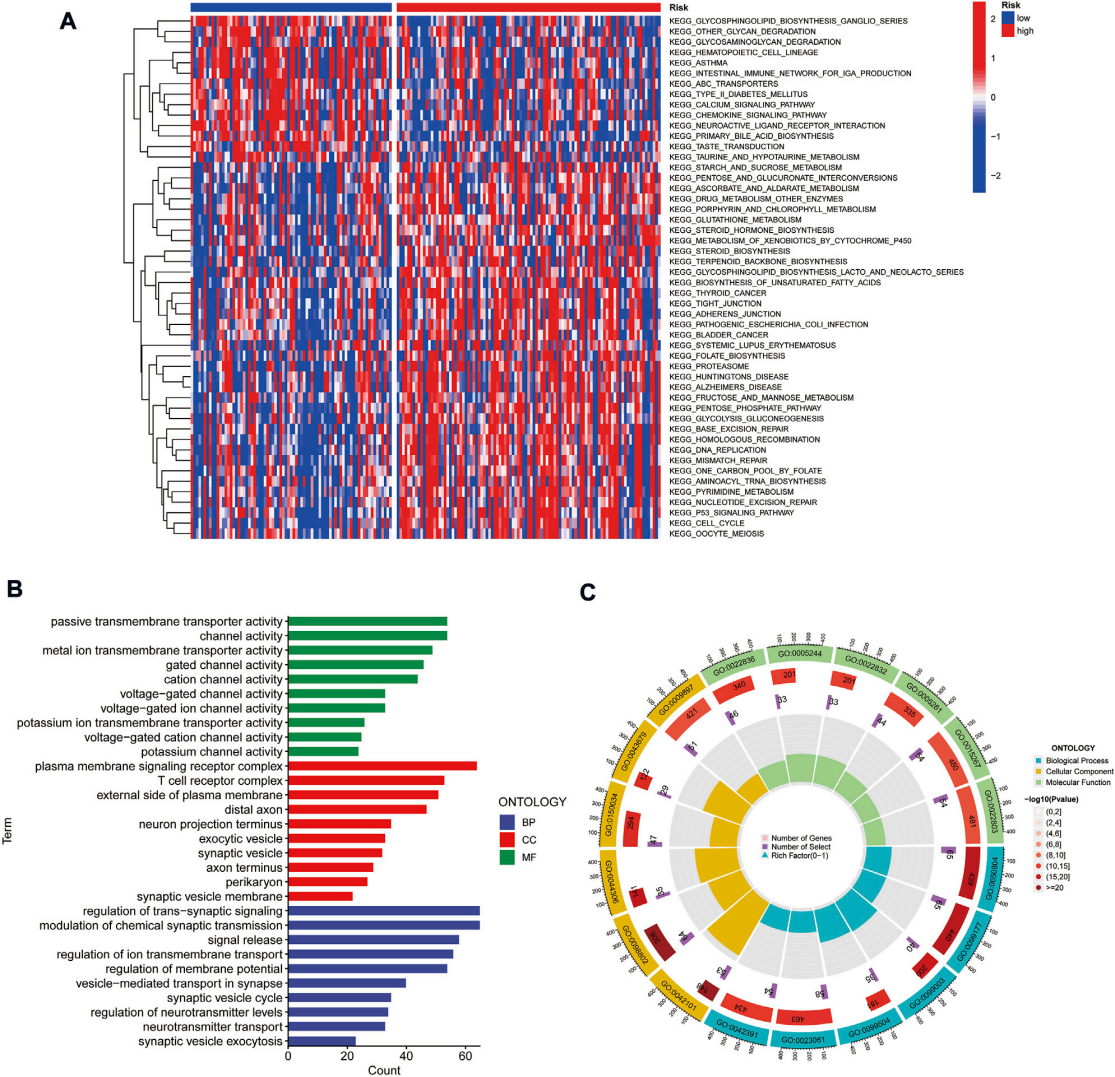
Gene set variation analysis and gene ontology analysis. **(A)** Heat map highlighting the differences in functional pathways in high-risk and low-risk groups. **(B,C)** Exploring the enrichment of differentially expressed genes between high- and low-risk groups in terms of biological function by GO analysis.

To explore the differences in biological behavior between high- and low-risk groups, we used GSVA to investigate the differences in functional pathways between the groups. The pathways enriched in the high-risk group included the cell cycle, DNA replication, p53 signaling pathway, mismatch repair, and fatty acid metabolism, which were associated with tumor invasion. On the other hand, functions such as intestinal immune network, chemokine signaling pathway, and glycan degradation were enriched in the low-risk group (Figure 6A). We further analyzed the enrichment of differentially expressed genes (DEGs) in different risk groups in terms of biological functions by GO analysis. The results suggested that DEGs were enriched in functions such as cellular ion channels, membrane receptors, T-cell receptors, and transduction of signals (Figures 6B,C).

### Correlation of risk model with tumor mutation burden in pancreatic adenocarcinoma

**FIGURE 7:**
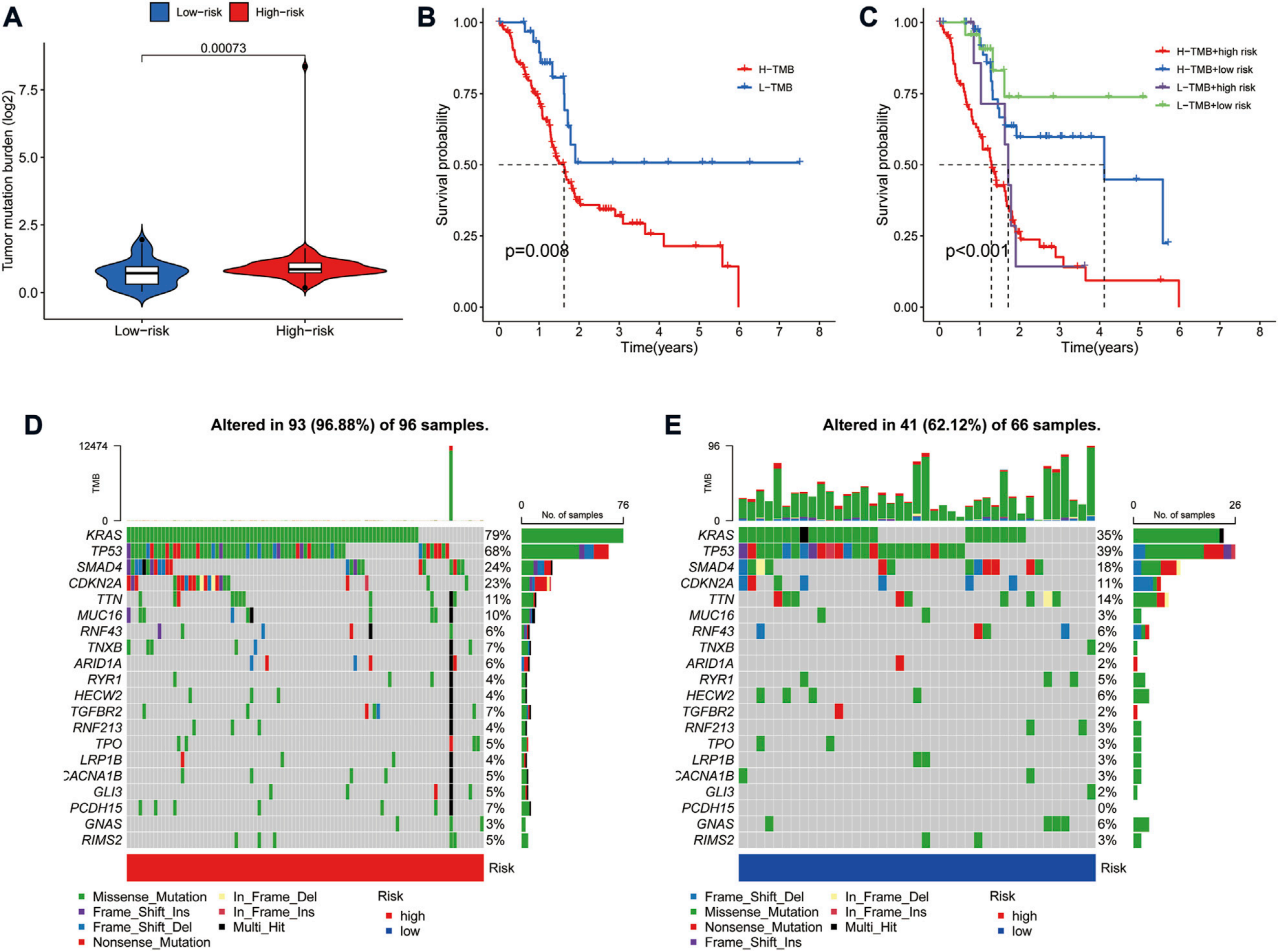
Correlation of risk model with tumor mutation burden in PAAD. **(A)** Violin plot of TMB status in the high- and low-risk groups **(B)** Kaplan Meier curve of high-TMB and low-TMB. **(C)** Kaplan-Meier curve of TMB + Risk. **(D)** Mutant gene waterfall plot in the high-risk group. **(E)** Mutant gene waterfall plot in the low-risk group.

Tumor mutation burden (TMB) is defined as the number of somatic mutations per megabase. TMB is a crucial driver in generating immunogenic neopeptides in tumor cells and affecting the patient’s response to ICIs (Sha et al., 2020). We used the TGCA somatic mutation data to generate TMB scores. Further analysis revealed that TMB levels were significantly lower in the low-risk group than in the high-risk group (Figure 7A). Also, survival analysis showed that higher TMB in PAAD was associated with a poorer OS (Figure 7B). Given the prognostic role of the risk model and TMB in PAAD, we further explored the prognostic value of combining the two by dividing all samples into four groups: high-TMB/high-risk, low-TMB/low-risk, high-TMB/low-risk, and low-TMB/high-risk. The results showed a significant difference in survival between the four groups (*p* < 0.001), with patients with high-TMB/high-risk having the worst OS and those in the low-TMB/low-risk group having the best overall survival (Figure 7C). In addition, the frequency of mutations was higher in the high-risk group (96.88%) than in the low-risk group (62.12%) (Figures 7D,E). The highest mutation frequencies were found in KRAS (79%), TP53 (68%) and SMAD4 (24%) in the high-risk group, while the highest mutation frequencies in the low-risk group were in TP53 (39%), KRAS (35%) and SMAD4 (18%).

### Cancer-associated fibroblasts-related long non-coding RNAs signature for prediction of the immune microenvironment

**FIGURE 8:**
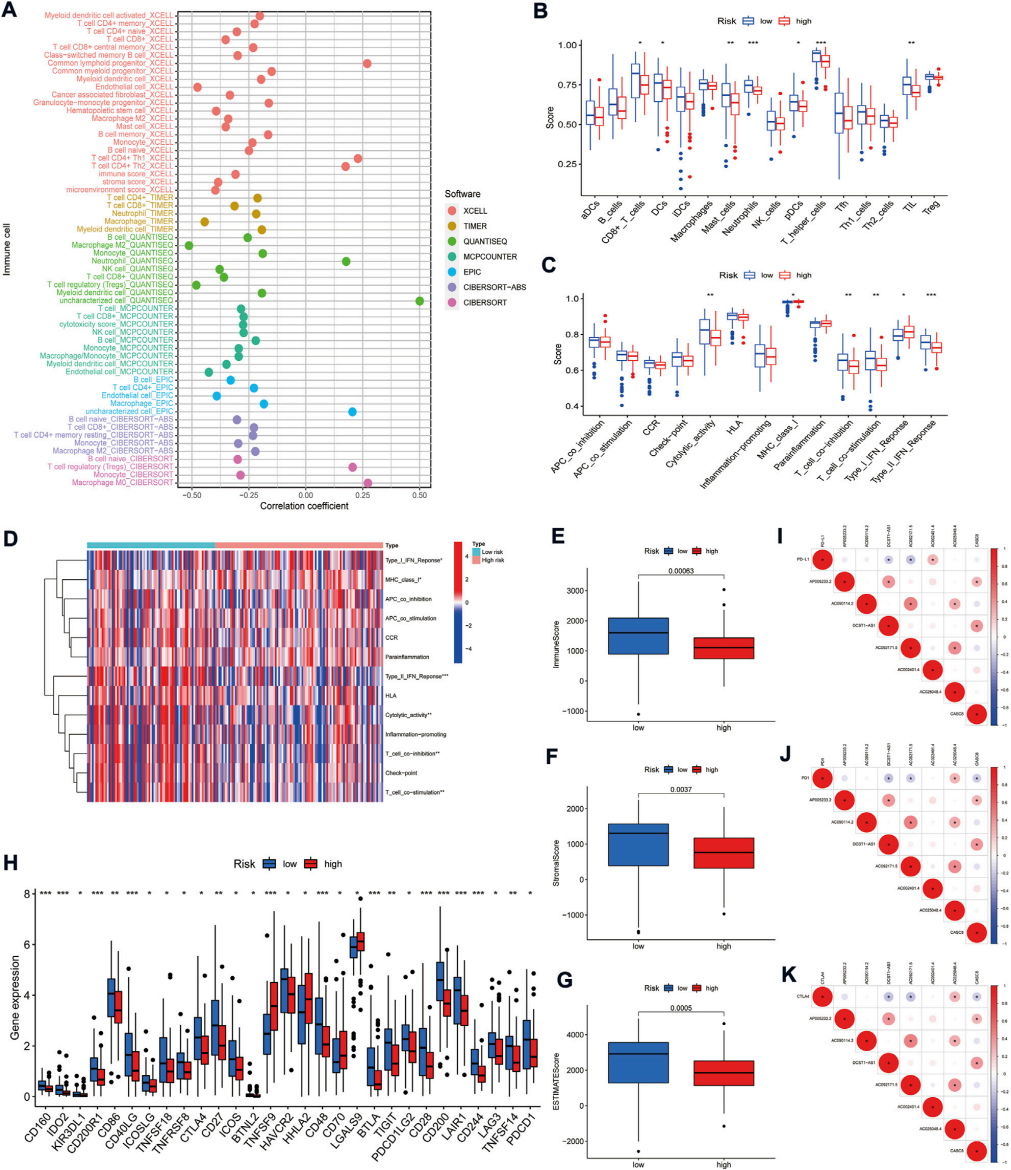
Correlation of the risk model with the immune microenvironment in PAAD. **(A)** Bubble plot of correlation coefficients between immune cells and risk scores. **(B)** Comparison of the enrichment scores of immune cells between high- and low-risk groups. **(C)** Comparison of the enrichment scores of immune-related functions between high- and low-risk groups. **(D)** Heat map depicting the status of immune-related functions in the high- and low-risk groups. **(E–G)** Correlation of high- and low-risk groups with immune cell score, stromal cell score, and ESTIMATE score. **(H)** Comparison of immune checkpoints in high- and low-risk groups. **(I–K)** Correlation between the expression of three immune checkpoints (PD-L1, PD-1, and CTLA4) and seven signature lncRNAs, respectively. *p < 0.05, **p < 0.01, and ***p < 0.001.

The tumor immune microenvironment (TIME) is closely related to the prognosis of patients with tumors. There is growing evidence that CAFs can synergize with TIME components, particularly immune cells, to render the tumor microenvironment (TME) immunosuppressive (Barrett R. and Puré E., 2020; Barrett R. L. and Puré E., 2020). To further explore the correlation between the CAFs-related lncRNAs signature and TIME, we analyzed the association between immune cells and risk scores. The results indicated that most immune cells were negatively related with the risk scores. In contrast, regulatory T cells (Tregs) on the CIBERSORT platform and common lymphoid progenitor, CD4^+^ Th1/2 cells on the XCELL platform, were positively correlated with the risk scores (*p* < 0.05) (Figure 8A). In addition, ssGSEA analysis showed that CD8^+^ T cells, dendritic cells (DCs), plasmacytoid dendritic cells (pDCs), neutrophils, mast cells, helper T cells, and tumor-infiltrating lymphocytes (TIL) were significantly fewer in the high-risk group than in the low-risk group (Figure 8B). In terms of immune-related functions, T cell co-stimulation, T cell co-inhibition, type II interferon (IFN) responses, and cytolytic activity were significantly weaker in the high-risk group than in the low-risk. The opposite trend was seen with the major histocompatibility complex (MHC) class I and type I IFN response (Figures 8C,D). Further, we analyzed the relationship between risk groups and ESTIMATE scores. The results suggested that the ESTIMATE score, stromal score, and immune score were significantly higher in the low-risk group than in the high-risk group (Figures 8E–G), which corroborated with the above results and together indicated that the low-risk group had a higher immune cell infiltration status.

ICIs therapy offers a new tool for clinical cancer treatment by enhancing anti-tumor immune responses through a regulatory pathway of T cells (Sharma and Allison, 2015). However, immune checkpoint therapy benefits only a small proportion of patients with specific tumor types, and one of the main problems is the lack of validated prognostic biomarkers (Sharma et al., 2021). The current ICIs target the programmed death-1 (PD-1), programmed death-ligand 1 (PD-L1), and cytotoxic T-lymphocyte-associated protein 4 (CTLA-4) ((Sharma et al., 2021). Although the expression of PD-L1 did not differ in the high- and low-risk groups, the mRNAs of most other immune checkpoint-related genes, including PD1 and CTLA4, were highly expressed in the low-risk group (Figure 8H). Our results suggest that low-risk patients may benefit more from ICIs. Finally, we analyzed the correlation of the expression between the seven lncRNAs and the three immune checkpoints. The results showed that DCST1-AS and AC092171.5 were negatively correlated with the expression of all three immune checkpoints (*p* < 0.05, Figures 8I–K). Together, these results suggest that CAFs-related lncRNAs signature might better distinguish PAAD patients with different tumor immune microenvironment characteristics and provide a basis for selecting clinical immunotherapy.

### The effect of risk score on the sensitivity of chemical compounds

**FIGURE 9:**
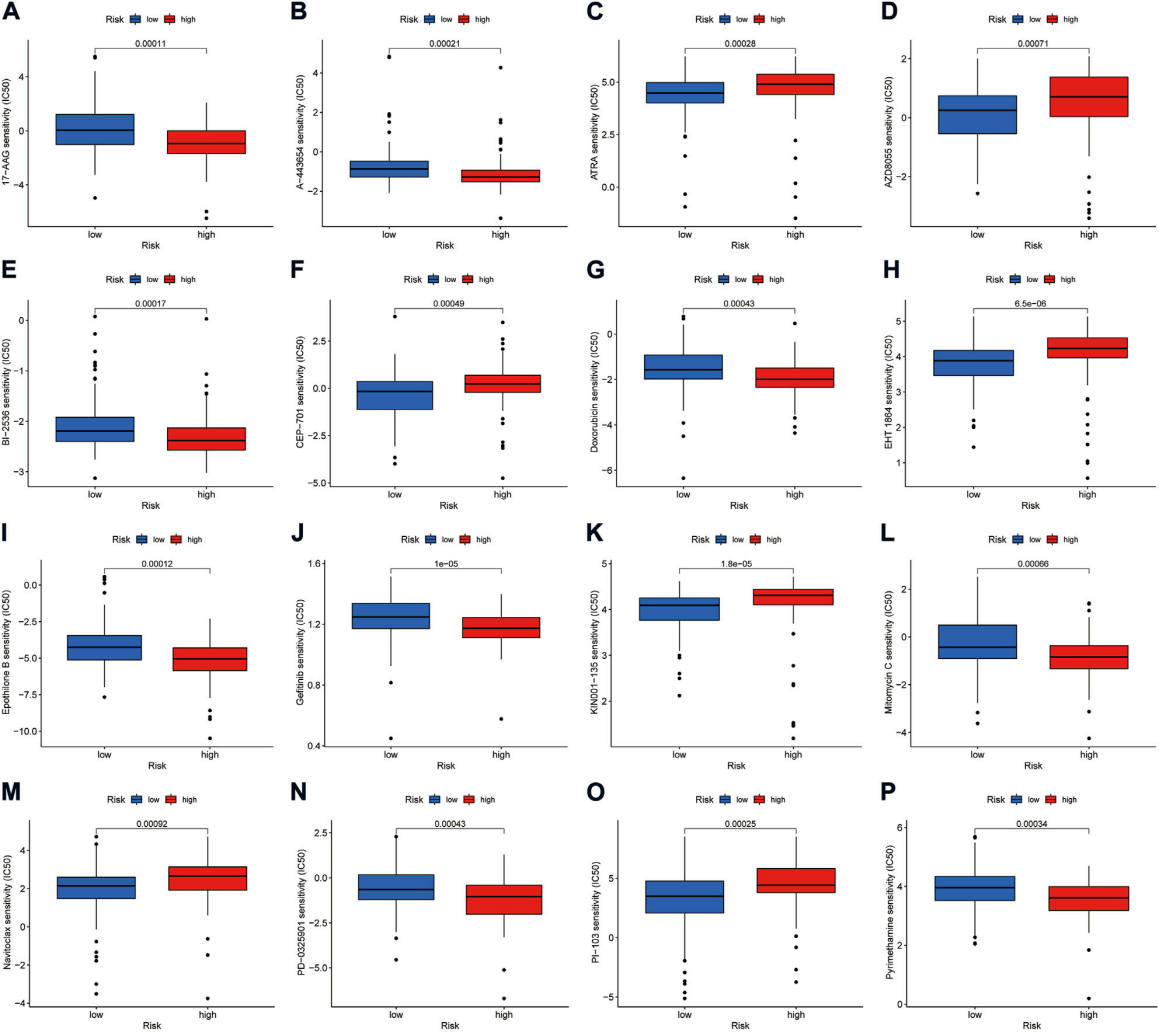
Investigation of drug sensitivity in risk groups. **(A–P)** Comparison of IC50 values for different agents in high- and low-risk groups.

Using the pRRophetic algorithm analysis, we found that the IC50 of some compounds differed between the high- and low-risk groups of the model (*p* < 0.001) (Figures 9A–P). Among them, the IC50 of mTOR inhibitor AZD8055, All-trans retinoic acid (ATRA), lestaurtinib (CEP-701), Bcl-2 family protein inhibitor navitoclax (ABT-263), and other drugs were higher in the high-risk group than in the low-risk group. The opposite effect was shown by drugs such as doxorubicin, gefitinib, and mitomycin C.

## Discussion

Dense mesenchyme formed by excessive fibrosis is an essential feature of TME in pancreatic cancers (Erkan et al., 2012). At the same time, fibrosis exacerbates the lack of vascularity and hypoxia in TME, which not only promotes tumor proliferation, invasion, and migration but also makes it resistant to anti-tumor agents. In addition, the low infiltration of effector T cells and high infiltration of immune suppressor cells in the TME of most pancreatic cancers makes them exhibit an immune-desert phenotype, i.e., an immunosuppressive TME phenotype (Muller et al., 2022). In this process, CAFs can recruit immunosuppressive cells, including myeloid-derived suppressor cells (MDSCs) and, through the induction of cytokines such as IL-6 and IL-11, and participate in tumor immune evasion mechanisms (Tang et al., 2012; Mace et al., 2013; Pothula et al., 2020). Moreover, the fibrous proliferative mesenchyme produced by CAFs also impeded the infiltration of effector immune cells into PAAD tumor areas (Ene-Obong et al., 2013; Wu et al., 2020). Collectively, these findings suggest an important role for CAFs in suppressive TIME.

lncRNAs broadly regulate the biological behavior of pancreatic cancer, such as promoting tumor angiogenesis, metastasis, proliferation, immune escape, and metabolic reprogramming (Chen et al., 2018; Deng et al., 2018; Guo et al., 2020; Hu et al., 2020; Zhai et al., 2021). Moreover, studies suggest that CAFs can regulate the function of lncRNAs (Ren J. et al., 2018; Ren Y. et al., 2018). Although lncRNAs have been shown to have a good predictive capability in PAAD prognosis (Yu et al., 2021; Zhu et al., 2022), the prognostic and immune microenvironmental predictive value of CAFs-associated lncRNAs in PAAD remains unclear.

In this study, we generated a CAFs-related lncRNA signature to predict the prognosis and immune microenvironmental landscape of PAAD patients. The results suggest that the risk score of this signature is an independent predictor of PAAD patients, and is also applicable to patients with different clinicopathological parameters. Together with the assessment of ROC and nomogram, it suggests that the constructed CAFs-related lncRNA signature can accurately predict the prognosis of PAAD patients. Among the seven lncRNAs used to construct the signature, AC092171.5 has previously been reported as an immune- and m6A-related lncRNA in pancreatic cancer and is significantly associated with patient prognosis (Wei et al., 2019; Cao et al., 2022). CASC8 has been suggested as a marker to predict the prognosis of PAAD and as a potential target for treatment (Wang et al., 2020; Ping et al., 2022), which is consistent with our findings. AC090114.2 was reported to be a pyroptosis-related lncRNA and was associated with PAAD prognosis and tumor immune microenvironment (Zhao et al., 2022). However, there are no related studies on the remaining lncRNAs in PAAD. Of these, AP005233.2 is thought to be associated with metabolism and patient prognosis in intrahepatic cholangiocarcinoma (Zou et al., 2021). DCST1-AS1 can enhance chemoresistance in triple-negative breast cancer cells by promoting TGF-β-induced epithelial-mesenchymal transition (Tang et al., 2020). In addition, AC025048.4 was identified as a ferroptosis-related lncRNA in lung adenocarcinoma (Zheng et al., 2021). Together, the above results suggest that the same lncRNA can be involved in regulating different biological functions. Given the prognostic value of these CAFs-associated lncRNAs in PAAD, their regulatory mechanisms in PAAD deserve further exploration.

In recent years, the advent of immunotherapy, represented by immune checkpoint inhibitors, has changed the goal of intervention in anti-cancer therapy, attempting to achieve tumor control by enhancing the host’s immune response (Galon and Bruni, 2019). However, the low overall response rate to immune checkpoint inhibitor therapy is currently a clinical challenge. One of the main reasons for this situation is the insufficient infiltration of effector T cells in tumors, referred to as “cold immune tumors" (Bonaventura et al., 2019; Galon and Bruni, 2019). In contrast, immunoinflammatory cancers characterized by high infiltration of CD8^+^ T cells and immune checkpoint activation are “hot immune tumors” (Galon and Bruni, 2019; Liu and Sun, 2021). The latter, in turn, is often the population that benefits from ICIs. Therefore, tumor immunophenotyping is vital for predicting the efficacy of immunotherapy in patients. In the present study, most immune cells, including CD8^+^ T cells, were negatively correlated with the risk score. ssGSEA analysis also validated this finding at both the immune cell and immune function levels. Further, ESTIMATE analysis suggested that the low-risk group had a significantly higher immune score, stromal score, and ESTIMATE score than the high-risk group, which corroborated with the results above and together suggested that the low-risk group had a higher immune cell infiltration status. These results partly explain the better prognosis of the low-risk group. Most immune checkpoint genes, including PD1, CTLA4, and LAG3, are highly expressed in patients in the low-risk group. It is further suggested that low-risk patients are more in line with the characteristics of “hot immune tumors” and might benefit from ICIs therapy more than high-risk patients.

TMB is thought to have a significant role in producing immunogenic neopeptides that are expressed on the MHC on the tumor cell surface and affect the patients’ responses to ICIs (Sha et al., 2020). Interestingly, our study showed a higher TMB in the high-risk group than in the low-risk group. However, pancreatic cancer has a low mutational burden compared to high mutational burden tumors such as melanoma (Alexandrov et al., 2013). From this perspective, TMB does not appear to be a good predictor of efficacy in PAAD ICIs. However, it is worth noting that in this study, TMB was significantly associated with prognosis, and its combination with risk score could more accurately predict the prognosis of PAAD patients. Our results also showed that the mutation rate of KARS and TP53 was much higher in the high-risk than in the low-risk group. Mutations in KRAS can impair T cell recognition of pancreatic cancer cells, leading to immune evasion (Cullis et al., 2018). As a well-known tumor suppressor gene, TP53 mutations can affect the recruitment and activity of T cells, which can also lead to tumor immune evasion (Blagih et al., 2020). These results suggest a high degree of immunosuppression in the high-risk group, leading to poorer survival.

Currently, the treatment of advanced PAAD is still dominated by chemotherapy, and almost all advanced patients experience disease progression even after treatment. And patients are recommended to be enrolled in clinical trials after second-line treatment. A crucial direction to the clinical trials is understanding how to carry out individualized combination therapy for patients. The CAFs-related lncRNA signature in this study provides the basis for selecting some chemotherapeutic and targeted drugs. ATRA has previously been shown to limit connective tissue proliferation and inhibit tumor growth in a PAAD model (Froeling et al., 2011). Meanwhile, ATRA can reverse the process in PAAD by which pancreatic stellate cells (PSCs) hinder the infiltration of effector immune cells into the tumor microenvironment (Ene-Obong et al., 2013). A phase II clinical trial of ATRA as a stromal-targeting agent in combination with chemotherapy for pancreatic cancer is underway (Kocher et al., 2020). This study suggests that the low-risk group is more sensitive to ATRA than the high-risk group. Our results indicate that the low-risk group is more susceptible to ATRA than the high-risk group. Furthermore, *in vitro* activation and extracellular matrix buildup of PSCs are suppressed by the Hsp90 inhibitor 17AAG (Peng et al., 2021). The IC50 values indicated that high-risk patients were more sensitive to 17AAG. Our data also suggest that high-risk patients are more susceptible to the chemotherapy drugs Doxorubicin, Pyrimethamine, and Mitomycin C than low-risk patients.

Although the signature generated in this research was validated by different methods, there remain some limitations. First, we only used TCGA database data for internal validation, whereas we still need to validate the signature in the future with a prospective large sample clinical cohort to test the applicability of the predictive signature. In addition, the mechanism underlying lncRNA association with CAFs in PAAD needs further experimental validation.

## Conclusion

In conclusion, the CAFs-associated lncRNAs signature identified in this study can effectively predict the prognosis and immune microenvironment profile of PAAD patients. It also provides a basis for understanding the possible mechanisms of the role of CAFs-related lncRNAs in PAAD and for clinical selection of ICIs, chemotherapeutic agents, and targeted drugs. Nevertheless, our findings require further validation in the future.

## Data Availability

The original contributions presented in the study are included in the article/Supplementary Material, further inquiries can be directed to the corresponding authors.
