# Supplementary material for: Construction of a cancer-associated fibroblasts-related long non-coding RNA signature to predict prognosis and immune landscape in pancreatic adenocarcinoma
Source: Front Genet. 2022 Sep 23;13:989719. doi: 10.3389/fgene.2022.989719 (PMC9538573; doi:10.3389/fgene.2022.989719)
Supplement: Supplementary file 1 [file DataSheet1.ZIP › Supplementary materials/List of Supplementary materials.docx]

# List of Appendixes

Appendix 1. 86 Cancer-associated Fibroblasts-related genes

Appendix 2. Gene mutation data in PAAD

Appendix 3. Tumor mutation burden data in PAAD

Appendix 4. Correlation data of CAFs-related genes and lncRNAs

Appendix 5. Combined data on expression and survival of CAFs-related lncRNAs

Appendix 6. Clinical data of PAAD samples in TCGA

Appendix 7. Univariate Cox regression analysis data

Appendix 8. Risk scores in the training, testing, and whole cohorts

Appendix 9. Risk coefficients of model CAFs-related lncRNAs

Appendix 10. Univariate and multivariate independent prognostic analysis data for the model

Appendix 11. Nomogram risk data

Appendix 12. Differentially expressed genes between high and low risk groups

Appendix 13. GSVA data

Appendix 14. GO analysis data

Appendix 15. ssGSEA data

Appendix 16. Correlation data between immune cells and risk scores

Appendix 17. Estimate scores

Appendix 18. Immune checkpoint-related genes

Appendix 19. CAFs-related gene expression data

Appendix 20. CAFs-related LncRNA expression data
